# Geospatial variations and determinants of contraceptive utilization among married reproductive age women in Ethiopia: spatial and multilevel analysis of Ethiopian Demographic and Health Survey, 2019

**DOI:** 10.3389/fgwh.2023.1151031

**Published:** 2023-09-22

**Authors:** Bewuketu Terefe, Mihret Getnet, Yonas Akalu, Yitayeh Belsti, Mengistie Diress, Yibeltal Yismaw Gela, Amare Belete Getahun, Desalegn Anmut Bitew, Daniel Gashaneh Belay

**Affiliations:** ^1^ Department of Community Health Nursing, School of Nursing, College of Medicine and Health Sciences, University of Gondar, Gondar, Ethiopia; ^2^ Department of Human Physiology, School of Medicine, College of Medicine and Health Sciences, University of Gondar, Gondar, Ethiopia; ^3^ Department of Epidemiology and Biostatics, Institute of Public Health, College of Medicine and Health Sciences, University of Gondar, Gondar, Ethiopia; ^4^ Department of Anesthesia, School of Medicine, College of Medicine and Health Sciences, University of Gondar, Gondar, Ethiopia; ^5^ Department of Reproductive Health, Institute of Public Health, College of Medicine and Health Sciences, University of Gondar, Gondar, Ethiopia; ^6^ Department of Human Anatomy, School of Medicine, College of Medicine and Health Sciences, University of Gondar, Gondar, Ethiopia

**Keywords:** multilevel analysis, spatial distributions, contraceptive, Ethiopia, married women

## Abstract

**Introduction:**

Contraception is the most effective method of preventing unwanted pregnancies and their associated disadvantages. It is critical to recognize one's desire to utilize contraceptives before drafting and implementing a good family planning program, especially in developing nations like Ethiopia.

**Objective:**

This study aimed to identify the geospatial variations and determinants affecting the utilization of contraceptives among married reproductive age women in Ethiopia.

**Method:**

This study was based on an extensive national survey, the Ethiopian Demographic and Health Survey. A total weighted sample of 5,743 married reproductive-age women was included. Because of the hierarchical nature of the DHS data, a spatial analysis multilevel logistic regression model was used to study individual and community-level factors that may influence contraceptives. The Bernoulli model was used by applying Kulldorff methods using the SaTScan software to analyze the purely spatial clusters of contraceptive usage. ArcGIS version 10.3 was used to visualize the distribution of contraceptives. A 95% confidence interval and a *p*-value of less than 0.05 were used to declare statistical significance.

**Result:**

The overall utilization of contraceptives was discovered at 41.25% (39.98, 42.53). Participants age range of 25–34 years [AOR = 0.80, CI: (0.66, 0.96,)] and 35–49 years [AOR = 0.50, CI 95%:(0.66, 0.96)] times less likely to use contraceptives than 15–24 years old respectively. Having primary [AOR = 1.47, CI 95%: (1.25, 1.73)], secondary [AOR = 1.42, CI 95%: (1.09, 1.83)] and higher education level [AOR = 1.92, CI 95%: (1.41, 2.60)], middle wealth [AOR = 1.48, CI 95%: (1.14, 1.90)], richer [AOR = 1.41, CI 95%: (1.07, 1.86)] and richest [AOR = 2.17, CI 95%: (1.52, 3.11)], having 1–4 ANC follow up have [AOR = 1.60, CI 95%: (1.26, 2.03)], gave birth at age of 35–44 [AOR = 0.29, CI 95%: (0.22, 0.37)], having 3–5 children [AOR = 1.26, CI 95%: (1.03, 1.52)], being from community of high level women education [AOR = 1.61, CI 95%: (1.21, 2.15)] were associated positively. Participants from Amhara, Oromia, Benishangul and SNNPR regions have revealed [AOR = 2.40, CI 95%: (1.53, 3.77)], [AOR = 1.64, CI 95%: (1.05, 2.56)], [AOR = 1.62, CI 95%: (1.01, 2.62)] and [AOR = 2.04, CI 95: (1.31, 3.19)], in contrast, Somali and Afar regions have shown [AOR = 011, CI 95%: (0.05, 0.22)] and [AOR = 0.31, CI 95%: (0.18, 0.54)] times less likely to use contraceptive services than Tigray Region respectively. The spatial analysis of contraceptive usage discovered that the northern, central and southern parts of the country had higher utilization of contraceptives than the eastern and northeastern of the country.

**Conclusion:**

The study revealed that contraceptive usage among married women is comparatively low, with wide regional variation. Raising awareness among mothers about the importance of antenatal care and assisting mothers who are financially disadvantaged or do not have access to health facilities will aid in providing better family planning services. Improving contraceptive information dissemination at community and regional levels is key to averting potential barriers.

## Introduction

Family planning (FP) and contraception is a procedure that mainly requires a discussion of agreement between a woman and a man. A trained FP service provider focuses on family health and the couple's desires to either limit or space their children (1) Contraception methods play a significant role in reducing the complication of child and maternal morbidity and mortality by unintended preventive pregnancies, including social costs (2, 3). This concept is also supported by the sustainable development goal (SDG) that emphasizes securing healthy lives and advocating well-being for everybody of all ages (3–5). Among the 1.9 billion Women of the Reproductive Age group (15–49 years) worldwide in 2019, 1.1 billion require family planning; of these, 842 million are using contraceptive methods, and 270 million have an unmet need for contraception (6, 7). Another report by the world health organization (WHO), 2017, estimated that there were 214 and 23 million reproductive-age women with the unmet need to use contraception methods and unintended pregnancies due to low-quality service, cultural opposition, and limited access and experience of side effects (8). The United Nations report shows that contraception use among women has been increasing in all world regions; however, sub-Saharan Africa is in the lower range (9). The utilization of contraceptive methods among women 15–49 years in middle and low-income countries reached 55% in 37 countries; however, below 20% in 23 countries, including Ethiopia in 2019 (10). Reports indicated that modern contraception methods prevented about 308 million unintended pregnancies, and 67 million unintended pregnancies could also be averted if the unmeet issue was solved (11).

Various studies have been investigating contributing factors of contraceptive use in sub-Saharan countries. These include poor quality of service and long distances between the health facilities and users' home (12–14). Studies also found that many individual and community level factors, such as age, marital status, religion, household wealth index, joint contraception decision, place of residence, employment status, community education level, and the number of children are associated with contraceptive use in various countries (13, 15–20). Women's knowledge, perception, and information exposure/reading newsletters and listening to the radio about contraceptive advantages were also associated factors (13, 18, 20, 21).

Pieces of evidence from the Ethiopian Demographic and Health Survey 2011 and 2016 (EDHS) depicted that factors such as educational attainment, number of living children, exposure to mass media, employment status, positive attitudes, and information about contraceptive methods were factors associated positively with contraceptive methods (22–24). However, older age, residence type, religion, regions of Afar, and Somali are associated negatively with contraceptive use (22, 24, 25). From this one can notice that there is a variation of modern contraceptive utilization in Ethiopia based on their regions, residences and other community and individual level factors. We will assess this variation for further validation.

According to several studies and analyses, investing in sexual and reproductive health in the geographical context remains a top policy priority, and joint investments in contraceptive, maternal and child health would help to address the problem (26–28). The Ethiopian federal ministry of health office created a strategy in 2015 to increase the use of contraceptives from 42% to 55% from 2016 to 2020 as part of the overall health sector transformation plan by the end of 2020 (29). The prevalence of contraceptive methods used in Ethiopia varies significantly from region to region, time to time and from study to study. For instance, data collected in 2011 and 2016 in EDHS shows the prevalence of contraceptive method utilization is 29.2% and 44.11%, respectively (22, 30). This data shows an escalation of contraceptive method use from 2011 to 2016 in Ethiopia. A systematic review study reports that long-acting and permanent contraceptive methods pooled prevalence among married women was 16.64% (31). On the other hand, a community-based survey conducted in Amhara region zonal towns showed that the overall majority of the modern contraceptive method was 38.9% (32); in western Ethiopia at Wolaita Soda and Ariba Minch towns, the prevalence was 70% and 63.4% respectively (24, 33), Oromia Region 18.2% (34), in Axum town 48.0% (35). The investigations mentioned above had concluded different overall prevalence rates; hence they are limited to a specified study area. The prevalence results of the studies mentioned above on modern contraceptive methods in different places and times in Ethiopia show similar findings, some of which are entirely different. It was found to be EDHS that could give us general information about the country to reconcile this contradiction, so it was necessary to analyze further the EDHS of 2019.

Despite this, the majority of studies in the country focused on contraception use, side effects, and socioeconomic variables. There was limited up-to-date information available in the country about the distribution of contraceptive techniques and their spatial mapping. To further the understanding of policy-makers and program planners, it is envisaged that current research on the state of contraceptive usage, unmet demand among married women, and the factors at individual and community levels would be crucial. The information would indeed guide the development of appropriate initiatives and programs to bridge the gaps in Ethiopia's women's underuse of contraceptive methods. In turn, this will make it possible for the nation to guarantee the accomplishment of Sustainable Development Goal three, which calls for ensuring that everyone has access to contraception and other sexual and reproductive health care. As a result, research that used contraceptive methods as an objective was only able to provide a general picture of where planning and policy decisions should be made. As a result, large-scale research that depicts contraception utilization status could give a useful piece of information for planning and policy decisions. As a result, the current study employed data from the EDHS 2019 to identify geospatial variations and determinants affecting the utilization of contraceptives among married reproductive age women in Ethiopia.

## Methods and materials

### Study design, period, and setting

A community-based cross-sectional study was employed in Ethiopia from March to June 2019 (36). Ethiopia is a country in the Horn of Africa found in East Africa (3°−14° N and 33°–48° E) with nine regional states [Afar, Amhara, Benishangul-Gumuz, Gambella, Harari, Oromia, Somali, Southern Nations, Nationalities, and People's Region (SNNP) and Tigray] and two city administrations (Addis Ababa and Dire Dawa). It has 68 zones, 817 districts, and 16,253 kebeles (the lowest administrative units of a country). It has a population of over 110 million. Of which, 39.81% of the people are less than 14 years with a 1:1 sex ratio to the general population. The country also has a death rate of 5.8/1,000, 22.2% of urbanization, with a very high degree of major infectious diseases (37, 38). This study used the recent Ethiopian Demographic and Health Survey 2019 (EDHS 2019) to determine the spatial distribution and determinants of modern contraceptive methods in Ethiopia. The Demographic and Health Survey (DHS) captures data on various aspects of women's health and well-being, including issues of interpersonal violence. The survey is a nationally representative study, with a weighted representative sample of 5,743 women aged 15–49. We retrieved the data for this study from the EDHS website www.dhsprogram.com after the request to access the approved and downloading allowed. Then 5,743 reproductive age (15–49 years) women who are married were pooled from the EDHS 2019 to conduct the utilization of contraceptives with geospatial variation analysis.

### Study population and sampling techniques

The EDHS sample was stratified and selected in two stages. Each region was stratified into urban and rural areas, yielding 21 sampling strata. In two stages, samples of Enumeration Areas (EAs) were selected independently in each stratum. Implicit stratification and proportional allocation were achieved at each lower administrative level by sorting the sampling frame within each sampling stratum before sample selection, according to administrative units at different levels, and using a probability proportional to size selection at the first stage of sampling.

In the first stage, 305 EAs (93 in urban areas and 212 in rural areas) were selected with probability proportional to EA size and independent selection in each sampling stratum. A household listing operation was carried out in all selected EAs from January through April 2019. The resulting lists of households served as a sampling frame for selecting households in the second stage. Some of the selected EAs for the 2019 Ethiopian Demographic and Health Survey (EDHS) were large, with more than 300 households. To minimize the task of household listing, each large EA selected for the 2019 EDHS was segmented. Only one segment was selected for the survey, with probability proportional to the segment size. Household listing was conducted only in the selected segment; that is, a 2019 EDHS cluster is either an EA or a component of an EA. In the second stage of selection, a fixed number of 30 households per cluster were selected with an equal probability of systematic selection from the newly created household listing (36).

### Data collection procedure, analysis and variables

The study was conducted based on EDHSs data by accessing the DHS program official database www.measuredhs.com after permission was received through an online request explaining the study's objective. The outcome variable with significant predictors was extracted from Ethiopia's Demographic and Health Surveys household data set. Data were extracted using STATA version 14.1, and Microsoft Excel was applied to prepare for the spatial analysis, and to compute the community-level factors. The final model of multivariable two-level mixed-effect logistic regression analysis included categorical variables with a *p*-value of less than 0.25 in bivariate two-level mixed-effect logistic regression analysis, where odds ratios with 95% confidence intervals were estimated to identify independent variables of modern contraceptive use. *p* values less than 0.05 were used to define statistical significance. The fixed and random effects were computed to examine individual and cluster variability. In addition, comprehensive information about the community and individual characteristics was provided. Model zero (the null model) which consists of only the outcome variable without any individual and community level factors, Model, I (a model with only individual-level variables), Model II (model with only community level factors), and Model III (a model with both the individual and community level factors) are the four models presented in this investigation respectively. Women were interviewed using questionnaires based on the DHS Program's standard questionnaires that were adapted to reflect the population and health issues relevant to Ethiopia, and several data from women were obtained. Socioeconomic and demographic information was also collected from women and households. Intention to use modern contraceptive methods among women of 15–49 age was used as a dependent variable. The independent variables were age, level of education, residence, wealth status, occupation, family size, exposure to media, sex, and region.

### Spatial analysis

Traditional regressions can be used to analyze data when the subjects in a study have a linear connection with the dependent variable. Put another way, the assumption of linearity is rarely met when data is structured, grouped, or hierarchical. Individual and community-level clusters differed in our scenario (hierarchies). We assumed multilevel logistic regression to account for variations because of the hierarchical nature of the data and inter-cluster variation. The differences within and across communities are considered in our research. Intra-community Correlation (ICC) was calculated using a community-level variation to determine the community effect. Following that, the model's fitness was evaluated using the Likelihood Ratio (LR) test, Median Odds Ratio (MOR), and Proportional Change in Variance (*p* We cross-tabulated the weighted frequency of dependent variables and cluster numbers to obtain the case to total proportion ratio to prepare data for spatial analysis (ArcGis). The data was then integrated with the results. We cleaned and eliminated data with zero Latitude/Longitude coordinates, and then used ArcGIS 10.7 to do spatial studies to see if the data pattern was concentrated, distributed, or random over the research area.

### Moran analysis

The spatial autocorrelation (Global Moran's I) was used to determine if patterns of use of contraceptive techniques in the study area are scattered, clustered, or random. Moran's, I output ranges from (−1) to (+1). Values close to −1 suggested a dispersed intention to use contraceptives, but values close to +1 indicated a clustered and distributed random inclination to use contraceptives.

### Hot spot analysis (Getis-Ord Gi* statistic)

The Getis-OrdGi* statistics were generated to see how the geographical autocorrelation of contraceptive method intention varies across Ethiopia. Hotspot analysis generates a Zscore and a *p*-value that instantly pinpoints the statistical significance of the clustering of the target variable across the study area at various significance levels. A “hotspot” (high intention to use contraceptive techniques) is indicated by a statistical output with a high GI*, while a “cold spot” is characterized by a statistical output with a low GI* (low intention to use contraceptive methods).

### Spatial interpolation

Because they incorporate spatial autocorrelation and statistically optimize the weight, ordinary Kriging and empirical Bayesian Kriging were used in this study. The usual Kriging spatial interpolation strategy was used to predict the intention to use contraceptive procedures in unobserved areas of the country.

### SaTscan statistics

For the local cluster detection, SaTScan Version 9.6 software was employed. A circular window that sweeps systematically throughout the study area was employed to find a substantial SaTScan clustering of contraceptive method intention. ArcGIS and SaTscan use comparable data preparation procedures, with the exception that SaTscan uses yes and no instead of proportion. Using relative risk (RR) and log-likelihood (LL), we presented the findings of major and secondary observed clusters.

### Study variables

#### Dependent variable

Current contraceptive use among married women of reproductive age is the dependent variable. It was defined as The Demographic and Health Survey defined the current use of modern contraceptive methods among married women as including male and female sterilization, intramuscular injections, intrauterine contraceptive devices (IUCD), contraceptive pills, implants, male condoms, lactational amenorrhea, and emergency contraception (39, 40). Women were divided into “users” and “non-users,” with “users” denoting those who used any contemporary contraceptive method and “non-users” representing those who did not use any contraceptive method or used Folkloric and Traditional methods.

#### Independent variables

The independent factors for contraceptive use were chosen based on previous research and the variable's availability in the 2019 EDHS dataset. Individual and community-level variables were aligned for a multilevel analytic method, and variables were broadly divided into two groups. Variables at the individual level Individual-level variables included women's age at the time of the survey, educational level, household wealth index, the total number of children born, exposure to mass media, experience antenatal care follow-up during pregnancy, health facility visits, place of delivery, region, residence, women community-level poverty, and education were included.

#### Community-level variables

The remaining variables are not accessible directly from EDHS, although the region and place of residence were considered community-level variables. As a result, we aggregated the variables at the community level based on individual data and then categorized the aggregated values as low or high if the mean values or cluster proportions were below or above the national average, respectively. Community-level women's education and community-level women's wealth status were all considered community-level variables as a result of this.

## Result

### Individual-level characteristics of study participants

A total of 5,743 weighted samples of married participants were enrolled in the current study. In the age group, 2,381 (41.45) participants were under 25–34 years old. In this study, nearly half, 2,979 (51.87), 2,203 (38.37), and 1,225 (21.34), were uneducated, orthodox in religion, and most affluent in wealth status, respectively. Most of the 5,578 (97.14) and 5,523 (96.18) have checked their baby at health facilities and know contraceptive methods, respectively, whereas nearly half of the 3,514 (61.19%) and 2,817 (49.06%) have given birth at the age of 10–19 years and follow more than four times of antenatal care follow up respectively (Table 1).

**Table 1:** Sociodemographic and other individual characteristics of married women in Ethiopia (*n* = 5,743).

Variables		Contraceptive current utilization		Total, *n* (%)
		No, *n* (%)	Yes, *n* (%)	
Age				
15–24		733 (53.18)	645 (46.82)	1,378 (23.99)
25–34		1,278 (53.67)	1,103 (46.33)	2,381 (41.45)
35–49		1,363 (68.69)	621 (31.31)	1,985 (34.56)
Religion				
Orthodox		1,146 (52.01)	1,057 (47.99)	2,203 (38.37)
Catholic		10 (34.42)	20 (65.58)	30 (0.53)
Protestant		840 (52.49)	760 (47.51)	1,600 (27.87)
Muslim		1,328 (72.36)	507 (27.64)	1,835 (31.96)
Traditional		42 (67.48)	20 (32.52)	62 (1.09)
Others		7 (63.63)	4 (36.37)	11 (0.19)
Highest educational level				
No-formal education		2,016 (67.66)	963 (32.34)	2,979 (51.87)
Primary		1,064 (51.19)	1,014 (48.81)	2,078 (36.19)
Secondary		195 (42.94)	258 (57.06)	453 (7.89)
Higher		100 (42.78)	133 (57.22)	233 (4.05)
The current age of a child				
0–2 years		1,423 (54.00)	1,213 (46.00)	2,636 (46.39)
More than two years		1,907 (62.59)	1,139 (37.41)	3,046 (53.61)
Household wealth Index				
Poorest		772 (73.15)	283 (25.85)	1,056 (18.38)
Poorer		723 (64.43)	399 (35.57)	1,122 (19.54)
Middle		618 (54.33)	519 (45.67)	1,137 (19.79)
Richer		674 (56.05)	529 (43.96)	1,203 (20.95)
Richest		586 (47.86)	639 (52.14)	1,225 (21.34)
Knowledge of any method				
No		219 (100)	0 (0.00)	219 (3.82)
Yes		3,154 (57.10)	2,369 (42.90)	5,523 (96.18)
Place baby first checked				
Home		69 (41.89)	95 (58.11)	164 (2.86)
Health facilities		3,305 (59.24)	2,273 (40.76)	5,578 (97.14)
Place of delivery				
Home		1,089 (62.60)	650 (37.40)	1,739 (30.28)
Health facility		2,285 (57.07)	1,719 (42.93)	4,004 (69.72)
Does the household have a				
Television	No	2,855 (60.60)	1,857 (39.40)	4,712 (82.92)
	Yes	475 (48.95)	496 (51.05)	971 (17.08)
Radio	No	2,456 (60.74)	1,587 (39.26)	4,043 (71.15)
	Yes	874 (53.35)	765 (46.65)	1,639 (28.85)
Telephone	No	3,281 (58.68)	2,310 (41.32)	5,591 (98.39)
	Yes	49 (54.11)	42 (45.89)	91 (1.61)
Number of ANC follow up				
No ANC follow up		660 (71.61)	262 (28.39)	922 (16.06)
1–4 ANC follow up		998 (49.85)	1,005 (50.15)	2,003 (34.88)
More than four follow up		1,714 (60.86)	1,103 (39.14)	2,817 (49.06)
Age of respondent at first birth				
10–19		2,014 (57.30)	1,500 (42.70)	3,514 (61.19)
20–34		907 (55.77)	719 (44.23)	1,626 (28.31)
35–44		453 (75.16)	149 (24.84)	602 (10.49)
Number of total children				
0–2		1,210 (52.50)	1,095 (47.50)	2,305 (40.13)
3–5		1,044 (55.94)	822 (44.06)	1,866 (32.49)
6 and above		1,120 (71.22)	452 (28.78)	1,572 (27.38)
Number of health facility visits				
1–2		3,071 (58.44)	2,184 (41.56)	5,255 (91.50)
3–5		303 (61.98)	185 (38.02)	488 (8.50)

### Community-level factors and characteristics of study participants

The majority, 4,174 (72.69%) of the participants, were from rural residencies. At the same time, 2,240 (39.01%), 2,834 (49.35%), and 2,579 (44.91%) of the participants were from the Oromia region, a community with low education level and low community female wealth status, respectively (Table 2).

**Table 2:** Community-level variables: descriptive result of contraceptive utilization among married women among contraceptive user women in Ethiopia (*n* = 5,743).

Variables	Contraceptive current utilization		Total, *n* (%)
	No, *n* (%)	Yes, *n* (%)	
Place of residence			
Urban	804 (51.24)	765 (48.76)	1,569 (27.31)
Rural	2,570 (61.56)	1,604 (38.44)	4,174 (72.69)
Region			
Tigray	224 (62.84)	133 (37.16)	357 (6.21)
Afar	56 (87.22)	8 (12.78)	64 (1.11)
Amhara	661 (50.81)	640 (49.19)	1,301 (22.66)
Oromia	1,335 (59.62)	905 (40.38)	2,240 (39.01)
Somali	271 (96.59)	10 (3.41)	281 (4.89)
Benishangul	40 (60.99)	26 (39.01)	66 (1.15)
SNMP	637 (54.79)	525 (45.21)	1,162 (20.23)
Gambela	16 (66.10)	8 (33.90)	24 (0.42)
Harari	11 (67.78)	5 (32.22)	16 (0.28)
Addis Ababa	98 (49.58)	99 (50.42)	197 (3.43)
Dire Dawa	24 (70.05)	11 (29.95)	35 (0.60)
Community-level women’s education			
Low	1,898 (66.83)	936 (33.17)	2,834 (49.35)
High	1,459 (50.33)	1,450 (49.67)	2,909 (50.65)
Community-level women’s wealth status			
Low	1,742 (67.55)	837 (32.45)	2,579 (44.91)
High	1,615 (51.04)	1,549 (48.96)	3,164 (55.01)

### Spatial distributions and hot spot analysis of contraceptive utilization

**Figure 1:**
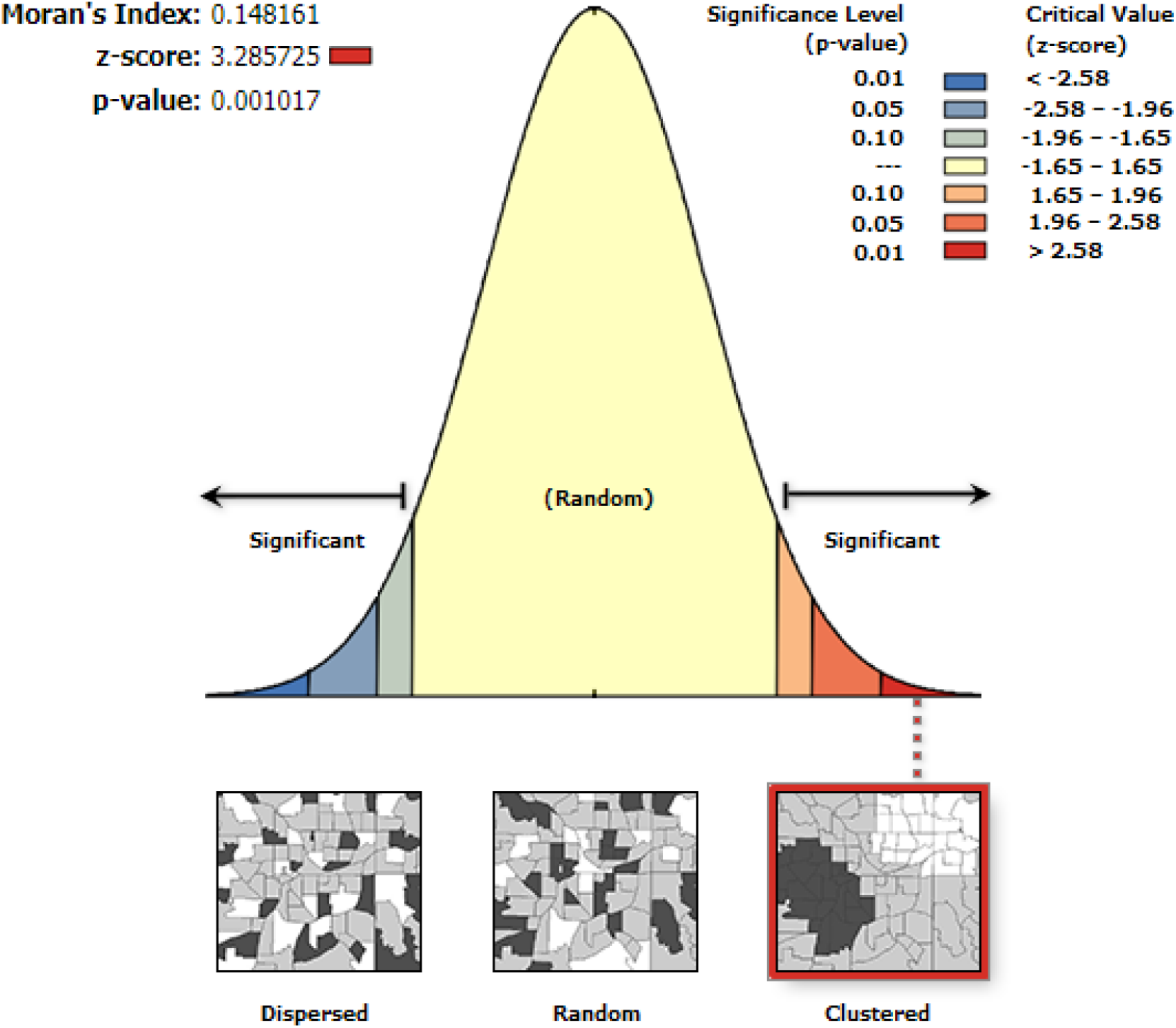
Spatial autocorrelation utilization of contraceptive methods among married women in Ethiopia, EDHS 2019.

This study depicted that the spatial distribution of contraceptive utilization was spatially clustered in Ethiopia with Global Moran's I 0.148 (*p* < 0.001). A cluster of high usage was observed. The outputs were automatically generated keys on each panel's right and left sides, respectively. Given the *z*-score of 3.28, there is less than a 1% likelihood that this clustered pattern could result from chance. The bright red and blue colours of the end tails indicate an increased significance level (Figure 1).

**Figure 2:**
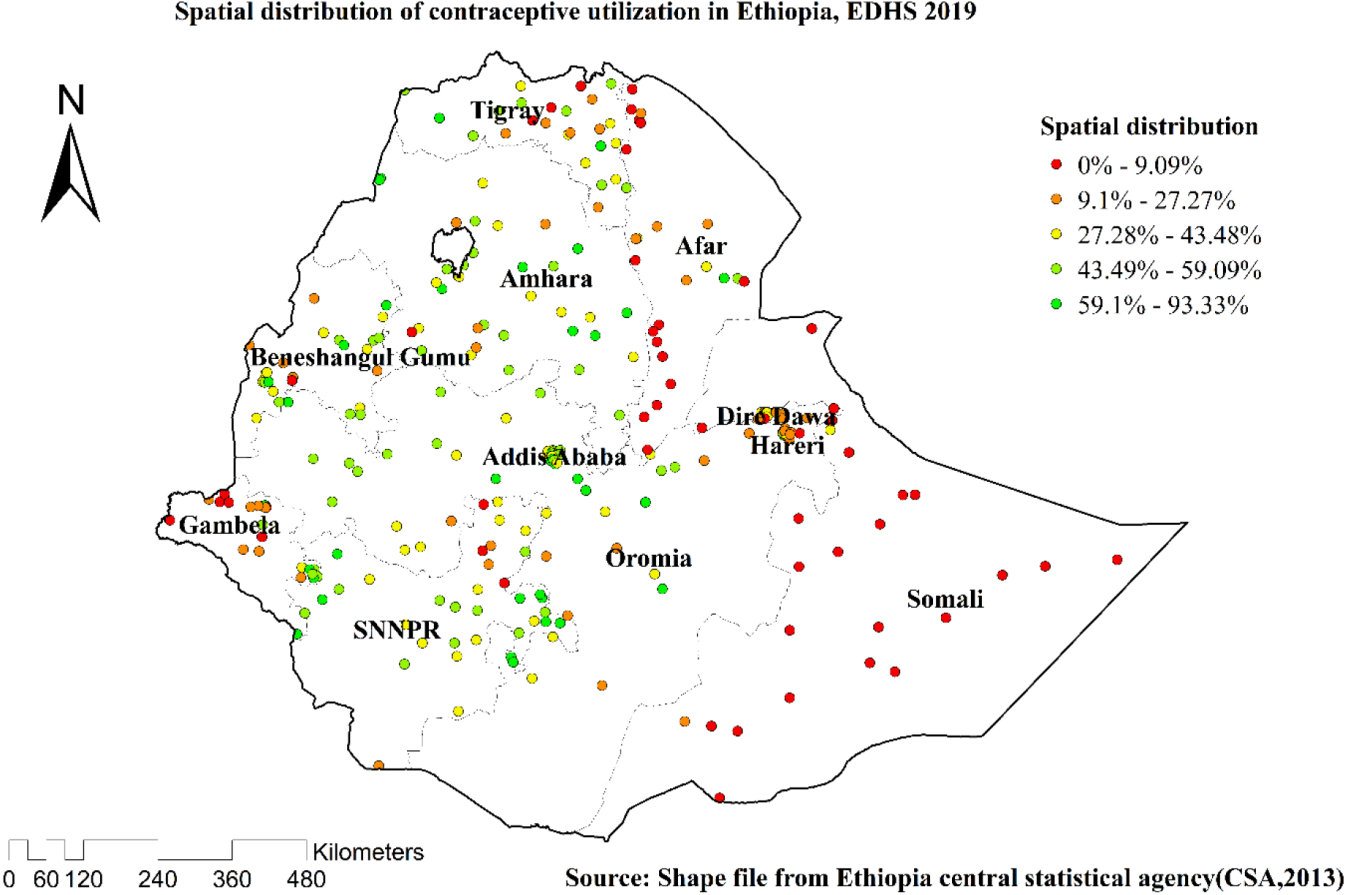
Spatial distributions of the utilization of contraceptive methods among married women in Ethiopia, EDHS 2019.

**Figure 3:**
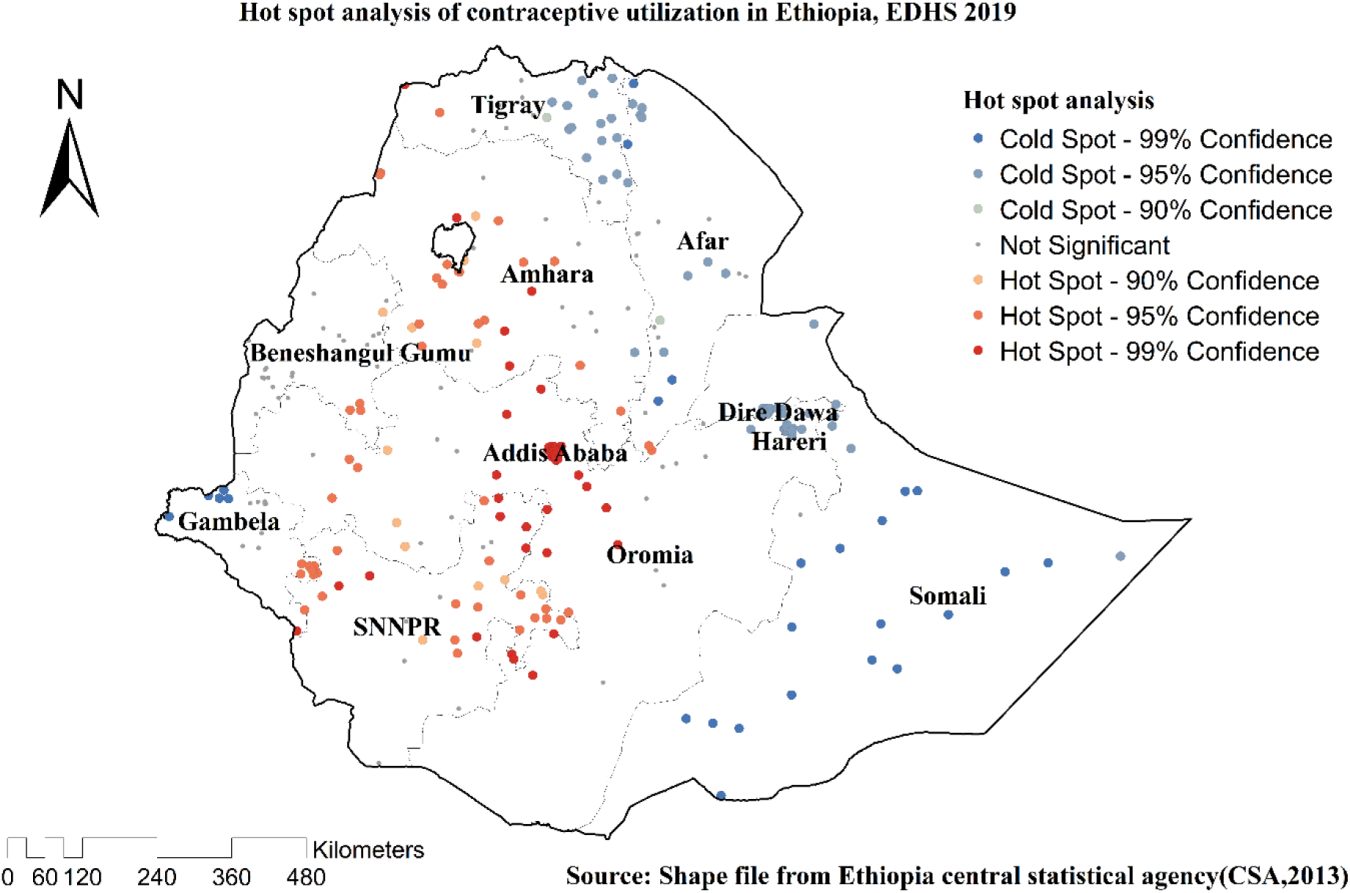
Hot spot analysis of the utilization of contraceptive methods among married women in Ethiopia, EDHS 2019.

Spatial clustering of contraceptive utilization was found at regional levels. Of 5,743 households interviewed in 2019, only 2,369 (41.26%) had received contraceptives. The highest health insurance coverage was spatially clustered in Amhara, Tigray, Oromia, and SNNPR regions. In contrast, Somali, Afar, and Dire Dawa regions had the lowest contraceptive employment (Figures 2, 3).

### Ordinary Kriging interpolation

**Figure 4:**
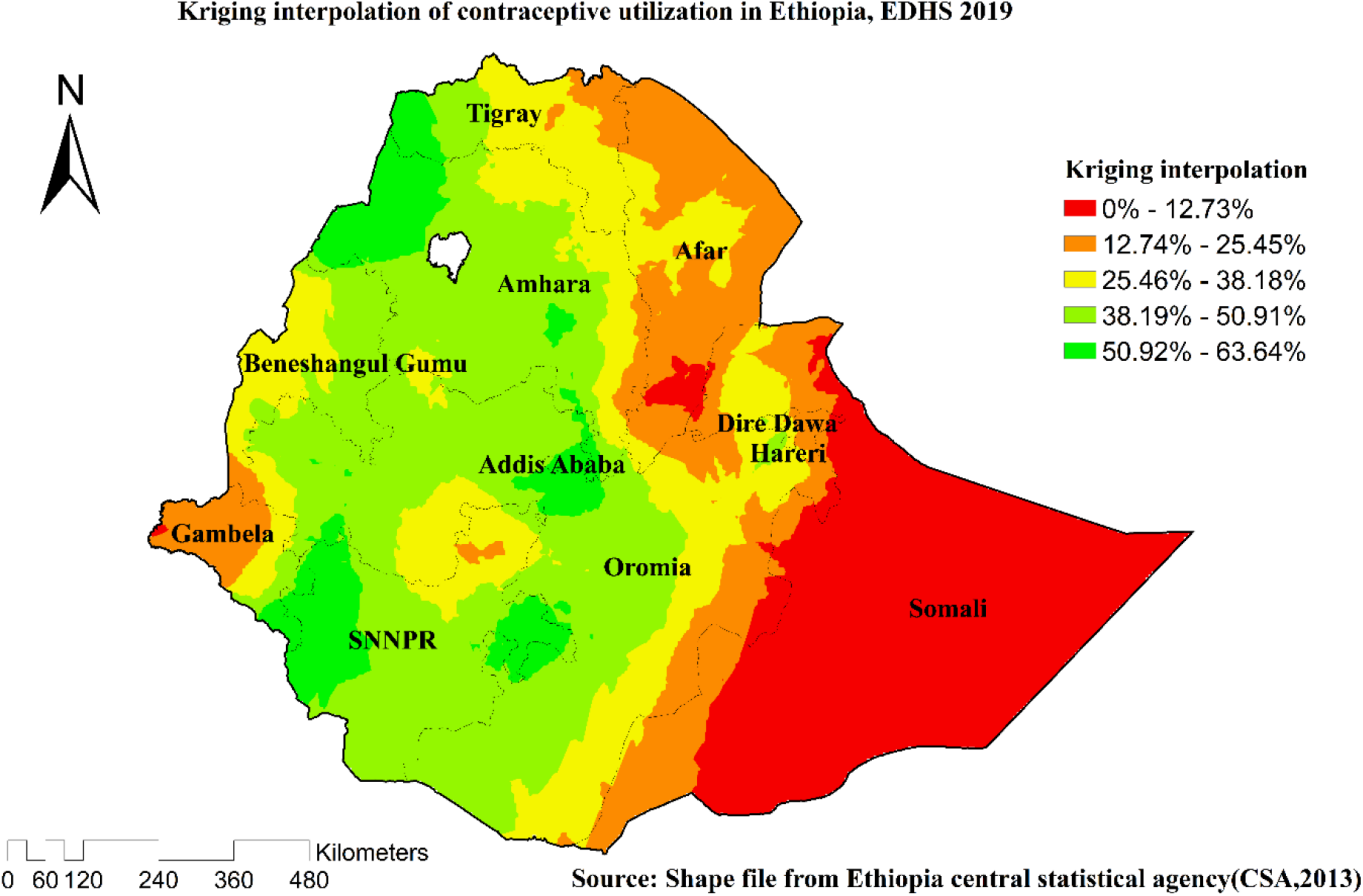
Kriging interpolation analysis of the utilization of contraceptive methods among married women in Ethiopia, EDHS 2019.

The highest prevalence of contraceptives was found in Amhara, SNNPR, Tigray, Addis Ababa, Benishangul, and areas of Oromia and Harari, according to geostatistical analysis. Ordinary Kriging interpolation calculates the distance between a known location and unknown areas to determine the facts in the event occurrence ranges. The measurements revealed a significant (Figure 4) site where the event could occur (prediction).

### SaTscan statistics

**Figure 5:**
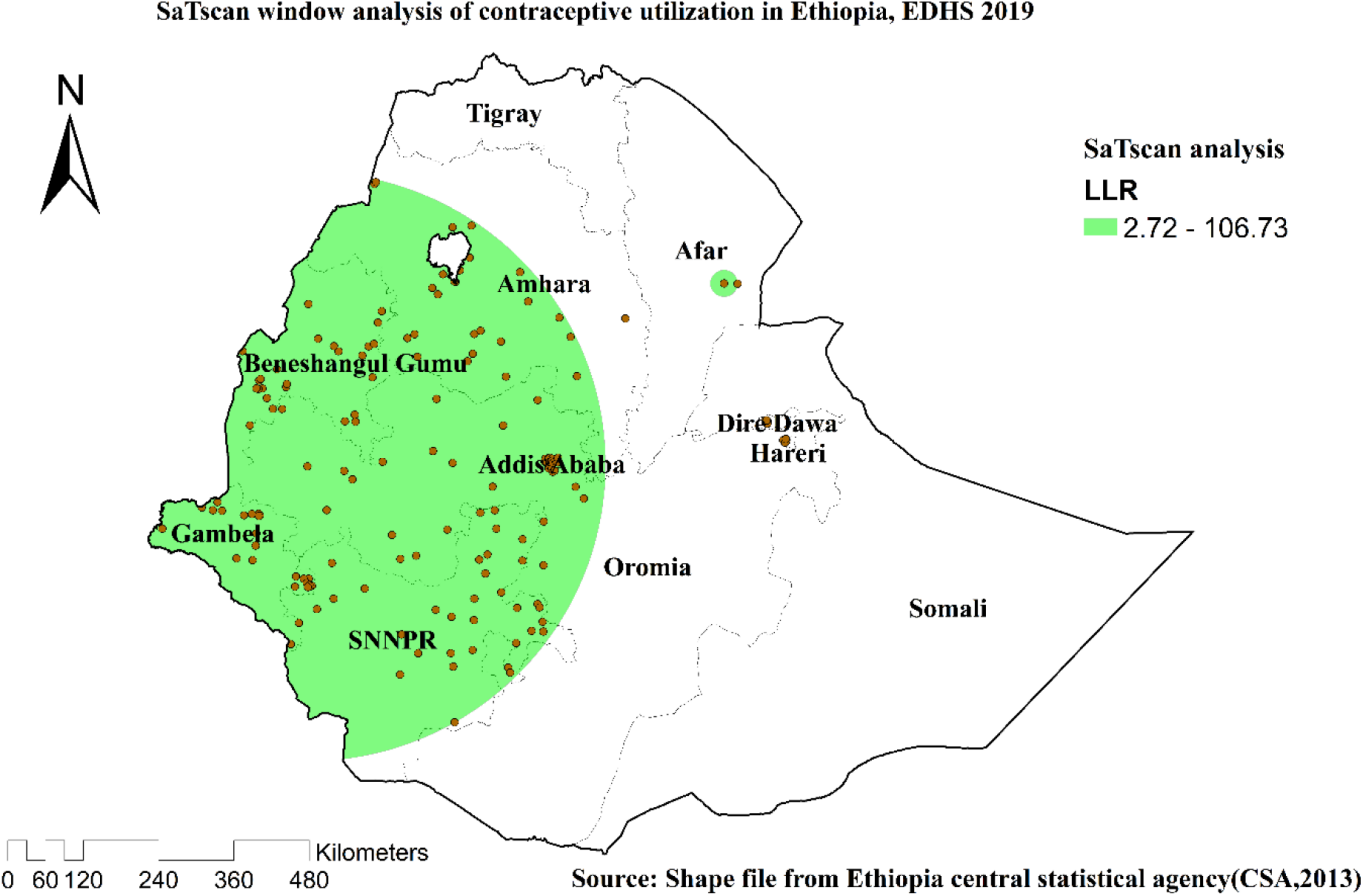
SaTscan scan statistics of the utilization of contraceptive methods among married women in Ethiopia, EDHS 2019.

EDHS 2019 revealed one significant local cluster area on the primary SaTscan window cluster. A total of 153 locations [coordinates/radius (8.944357 N, 35.279628 E)/468.49 km] with RR of 1.72 and LL of 106.73 was found in the primary SaTscan window cluster. The regions included were Amhara, Benishangul, Addis Ababa, Gambella, and SNNPR, with very few parts of the region (Table 3, Figure 5).

**Table 3:** Significant spatial clusters with high-rate contraception utilization among married women in Ethiopia, EDHS, 2019.

Cluster	Enumeration areas (Cluster detected)	Coordinates (radius)	Population	Cases	RR	LLR	*p*-value
1	118, 92, 120, 94, 169, 86, 168, 155, 207, 208, 209, 167, 170, 211,93, 154, 230, 156, 212, 150, 153, 152, 147, 213, 229, 218, 151, 157,194, 149, 217, 97, 214, 164, 225, 221, 226, 220, 206, 227, 222, 161,223, 210, 224, 228, 158, 166, 98, 160, 96, 146, 148, 195, 201, 163,91, 200, 87, 119, 95, 77, 215, 219, 162, 80, 159, 79, 165, 174, 216,112, 52, 171, 204, 196, 179, 176, 99, 72, 177, 180, 191, 76, 189, 53,73, 173, 75, 70, 205, 190, 192, 71, 178, 203, 74, 259, 198, 81, 54,262, 100, 260, 261, 257, 258, 274, 275, 276, 263, 265, 256, 277, 175, 264, 270, 279, 266, 267, 273, 278, 280, 271, 197, 268, 59, 199, 272,269, 184, 116, 57, 101, 187, 185, 115, 60, 90, 182, 84, 181, 67, 172,58, 186, 188, 65, 82, 51, 85, 55, 202	(8.944357 N, 35.279628 E)/468.49 km	2,870	1,250	1.72	106.73	0.0001

### Random effect analysis and model comparison

In the first model (null model), the ICC showed that individual differences accounted for the remaining unexplained 72.33 percent of the overall variability for contraceptives, while variations between clusters/EA accounted for about 27.67 percent of that variability. Additionally, the median odds ratio showed that there was variation in the use of contraception among clusters. It was 2.87 in the null model, meaning that if women were randomly chosen from two distinct clusters, those with a higher probability of using contraceptives had a 2.87 times greater chance of doing so than those with a lower probability (EAs). The whole model described roughly 63.41 percent of the variation in contraceptives in terms of PCV. One IV was also chosen as the model that fit the user the best (which had the lowest deviance) (Table 4).

**Table 4:** Individual and community-level factors associated with contraceptive utilization among married/in-union women in Ethiopia (*n* = 5,743).

Variables	Null model	Model I	Model II	Model III
	AOR (95% CI)	AOR (95% CI)	AOR (95% CI)	AOR (95% CI)
Age				
15–24		1		1
25–34		0.84 (0.69, 1.02)		0.80 (0.66, 0.96)*
35–49		0.53 (0.41, 0.68)*		0.50 (0.38, 0.64)*
Highest educational level				
No-formal education		1		1
Primary		1.52 (1.29, 1.79)*		1.47 (1.25, 1.73)*
Secondary		1.49 (1.15, 1.92)*		1.42 (1.09, 1.83)*
Higher		2.03 (1.49, 2.77)*		1.92 (1.41, 2.60)*
Household wealth index				
Poorest		1		1
Poorer		1.62 (1.27, 2.07)*		1.25 (0.98, 1.59)
Middle		2.13 (1.65, 2.75)*		1.48 (1.14, 1.90)*
Richer		2.10 (1.61, 2.75)*		1.41 (1.07, 1.86)*
Richest		2.96 (2.20, 3.96)*		2.17 (1.52, 3.11)*
Place of delivery				
Home		1		1
Health facility		1.12 (.92, 1.36)		1.08 (0.86, 1.29)
Number of ANC follow up				
No ANC follow up		1		1
1–4 ANC follow up		1.74 (1.25, 2.02)*		1.60 (1.26, 2.03)*
More than four follow up		1.38 (1.06, 1.79)*		1.28 (0.98, 1.66)
Age of respondent at first birth				
10–19 years		1		1
20–34 years		1.06 (0.91, 1.24)		1.08 (0.92, 1.26)
35–44 years		0.28 (0.22, 0.36)*		0.29 (0.22, 0.37)*
Number of children				
0–2		1.19 (0.93, 1.53)		1.19 (0.93, 1.53)
3–5		1.27 (1.05, 1.55)*		1.26 (1.03, 1.52)*
6 and above		1		1
Number of health facility visit				
1–2		1		1
3–5		1.18 (0.93, 1.49)		1.15 (0.91, 1.46)
Place of residence				
Urban			1	1
Rural			0.81 (0.59, 1.10)	1.12 (0.79, 1.60)
Region				
Tigray			1	1
Afar			0.27 (0.16, 0.46)*	0.31 (0.18, 0.54)*
Amhara			1.99 (1.23, 3.08)*	2.40 (1.53, 3.77)*
Oromia			1.41 (0.92, 2.17)	1.64 (1.05, 2.56)*
Somali			0.06 (0.03, 0.14)*	0.11 (0.05, 0.22)*
Benishangul			1.44 (0.91, 2.29)	1.62 (1.01, 2.62)*
SNMP			1.73 (1.12, 2.65)*	2.04 (1.31, 3.19)*
Gambela			0.98 (0.61, 1.58)	0.98 (0.60, 1.60)
Harari			0.74 (0.46, 1.20)	0.70 (0.43, 1.16)
Addis Ababa			1.19 (0.71, 2.01)	1.21 (0.71, 2.07)
Dire Dawa			0.64 (0.39, 1.04)	0.67 (0.40, 1.11)
Community-level women education				
Low			1	1
High			1.31 (1.01, 1.69)*	1.11 (0.85, 1.45)
Community-level women wealth status				
Low			1	1
High			1.89 (1.46, 2.45)*	1.61 (1.21, 2.15)*
Random parameters and model comparison				
Community-level variance	1.27	0.87	0.43	0.45
ICC (%)	27.67	21.02	11.51	12.01
MOR (95% CI)	2.87 (2.57, 3.32)	2.43 (2.18, 2.76)	1.86 (1.71, 2.06)	1.89 (1.73, 2.10)
PCV (%)	Reference	28.86	65.20	63.41
LR	−3,353.36	−3,155.27	−3,226.57	−3,067.03
DIC (2LLR)	6,706.72	6,310.54	6,453.14	6,134.06
AIC	6,710.727	6,348.53	6,483.145	6,198.07

* Significant at *p*-value <0.05.

ICC, Intra cluster correlation, MOR, Median Odds Ratio, PCV, Proportional Change of Variance, DIC, Deviance information criterion, LLR, Log Likelihood Ratio, AIC, Akaike information criterion.

### Factors associated with contraceptive utilization

After adjusting the possible confounders, plenty of important determinant variables were statistically significant for the utilization of contraceptive methods at the individual and community levels. Participants whose age group was from 24 to 34 and 35–49 years old were [AOR = 0.80, CI: (0.66, 0.96)] and [AOR = 0.50, CI 95%: (0.66, 0.96)] times less likely to utilization contraceptive methods compared to mothers from 15 to 24 years old age respectively. Similarly, household wealth status and educational level were positively associated with contraceptive methods. Participants had primary [AOR = 1.47, CI 95%: (1.25, 1.73)], secondary [AOR = 1.42, CI 95%: (1.09, 1.83)] and higher [AOR = 1.92, CI 95%: (1.41, 2.60)] times more likelihood of positive tendency to utilize contraceptive than participants with no education respectively. Regarding wealth status in the same fashion, participants in the class of, middle have shown [AOR = 1.48, CI 95%: (1.14, 1.90)], richer [AOR = 1.41, CI 95%: (1.07, 1.86)] and richest [AOR = 2.17, CI 95%: (1.52, 3.11)] times more positive association than the poorest one respectively. Those mothers having 1–4 times antenatal care follow up during their pregnancy have [AOR = 1.60, CI 95%: (1.26, 2.03)] times to use contraceptives than did not have antenatal care follow up during their pregnancy period. On the other hand, mothers who have given their first birth at the age of 35–44 years old have [AOR = 0.29, CI 95%: (0.22, 0.37)] times less likely to be contraceptive users than mothers classified from 10 to 19 years old. Mothers whose children number is from 3-to 5 have [AOR = 1.26, CI 95%: (1.03, 1.52)] times more positive inclination to use contraceptives than mothers who have six and move children. Mothers from high levels of community-level women's education have shown [AOR = 1.61, CI 95%: (1.21, 2.15)] times probability of utilizing contraceptives. Participants from Amhara, Oromia, Benishangul and SNNPR regions have revealed [AOR = 2.40, CI 95%: (1.53, 3.77)], [AOR = 1.64, CI 95%: (1.05, 2.56)], [AOR = 1.62, CI 95%: (1.01, 2.62)] and [AOR = 2.04, CI 95: (1.31, 3.19)] times more tendency to utilize contraceptive methods than Tigray region respectively. In contrast, Somali and Afar regions have [AOR = 011, CI 95%: (0.05, 0.22)] and [AOR = 0.31, CI 95%: (0.18, 0.54)] times less likely to use contraceptive services than Tigray Region respectively (Table 4).

## Discussion

The present study has attempted to assess the current status of contraceptive method utilization among married reproductive-age women in Ethiopia. The current contraceptive utilization was found at 41.25% (39.98, 42.53) only. This finding is higher than studies done in Ethiopia in 2016 29.2% (22); however, it is lower than studies conducted in Ethiopia 63.4% (24) in 2017, and almost the same as a study done in Indonesia 38.5% (41) in 2020, and Nigeria 39.2% (42) in 2018. Because of the countries' profiles of family planning experience, participants' attitudes, knowledge, and educational backgrounds toward family planning desire, it's probable that the findings from this research are less than those of the previous one. On the other hand, this study discovered a considerably higher prevalence of contraceptive intention, which could be due to limiting confounding factors at both the individual and community levels, which could have positive or negative effects on contraceptive use. This research indicated that women's decisions to use contraceptives were based on individual characteristics with a small sample size and study environment. It's possible that this influenced their prevalence findings.

This study declared that age was a determinant factor of contraceptive methods. Participants aged 25–34 years and 35–49 years are less likely to use contraceptives in their future families than the 19–24 years old mothers. This study discovered the same finding as studies done in Malawi (43), Nigeria (44), Indonesia (45), and Ethiopia (22, 46). This could be explained by it being well known that as a mother gets older, her chances of having a baby decrease even if she needs and desire to use contraceptive may decrease. On the other hand, as mothers get older, their chances of finishing their education journey increase, and their desire to have a baby increases. They may also not use contraceptives because they are more likely to live by themselves and manage their income and way of thinking. They are also more likely to have their source of income. Another possibility is that this group consisted of women who were either done having children or desired to spread them out, in contrast to the second group, which consisted of women who had not yet started having children. Another factor would be that teens were less likely to seek family planning services as a result of societal expectations that they shouldn't engage in premarital sex. The low frequency of contraception among women between the ages of 15 and 24 is probably caused by the fact that the majority of these women participate in risky sex, are newlyweds, and believe that marriage should be centered on the institution of having children. For a young mother, it may be challenging to get access to contemporary family planning services (47, 48).

The study also figured out that participants with primary, secondary, and higher educational levels have more likelihood of a positive tendency to utilize contraceptives than participants with no education. This study declared similar findings to studies done in Nigeria (44), East Africa (49), and Ethiopia (22, 24, 46). Possible justification might include educated women who may have better information and a positive attitude about birth control compared to uneducated women. In addition, as these women learn, their ability to become more self-reliant will increase, and they will be able to cope with the harmful effects of society. Alternatively, these educated women may be residents of urban areas and enjoy more family planning services.

Mothers classified under the middle, richer and richest wealth status index have more likelihood of a positive tendency to utilize contraceptives than participants classified under poorest. This finding is in agreement with other studies done in Uganda (50), East Africa (49), and Ethiopia (46, 51). In countries like Ethiopia, it is common for husbands to dominate and have self-determination (52, 53). Therefore, better-off mothers have a greater right to self-determination than the poorest mothers to spend on transportation and use what they want to stay healthy. In other words, poor mothers may not have the information and may simply consider giving birth as an option and source of income generation. Although contraption is free of charge in Ethiopia, this could be due to the direct or indirect fees that women may spend in order to obtain contraception. Another explanation could be biases and misconceptions about contraception use. Women may assume that contraception is unsuitable for women who work hard, as poor women typically do. Another factor that has been linked to contraceptive use in previous studies is exposure to family planning messaging in the media (51, 54, 55).

Those mothers having 1–4 times antenatal care follow up during their pregnancy have shown a better tendency to use contraceptives than did not have antenatal care follow-ups during their pregnancy period. This judgment is incongruent with other investigations investigated in middle and lower-income countries (56). Mothers having antenatal care follow-up during pregnancy leads mothers to benefit more from a family planning package, as they are better informed and counselled by health professionals than unsupervised (45). On the other hand, they may have a better view of family planning (45).

On the other hand, mothers who have given their first birth at 35–44 years old are less likely to be contraceptive users than mothers classified from 10 to 19 years old. This statistically significant outcome came up similar to the results of studies done in Uganda (50). This is actual logic because these mothers did not give birth in the age range they were supposed to give birth to, and now they are too old to try to have a baby before they stop giving birth. As a result, women between the ages of 10 to 19 are more likely to use contraceptives than 35–44 women.

Mothers whose children number is from 3-to 5 have a higher positive inclination to use contraceptives than mothers who have six and move children. This outcome has gone similarly to studies done in Malawi (57), Iran (58), Ghana (59), Uganda (50), and Ethiopia (60). Mothers who have given birth to 3–5 children are more likely to have contraceptives than mothers who have given birth to six and more than six children due to their sociodemographic variance. Alternatively, mothers with six or more children may not be able to use contraceptives because they may be too old to give birth and desire not to have more children (24, 49, 53, 60).

Mothers from high levels of community-level women's education have revealed a higher probability of utilizing contraceptives than their counterparts. The study agreed with other literature conducted in Nigeria (44) and Uganda (50). When the individuals around her have a positive attitude toward modern contraception and have better quality and healthier kids, for example, the mother will get more outstanding bravery and self-confidence to use them. She might persuade others in her society that contraception is beneficial for her, her kid, and her family. Or, if she has a mistaken perspective of birth control, she is more likely to gain from it if most of the population benefits. Then quickly finds the information she requires.

Through her friends and neighborhood participants from Amhara, Oromia, Benishangul, and SNNPR regions, *p* has revealed more tendency to utilize contraceptives. In contrast, the Somali and Afar regions are less likely to use contraceptive services than the Tigray Region, respectively. This impressive result has made a similar conclusion to studies done in Ethiopia (22, 60). This could be explained by the Amhara, Oromia, Benishangul, and SNNPR regions, compared to others, which have the highest population density in Ethiopia, with a relatively well-developed population in economic, political, and awareness aspects of contraceptive methods and have better access, availability to medical care with the number of nearby health facilities, so they are more likely to use contraception. Besides these factors, policy makers and implementers in these regions might have accomplished a good achievement with great passion.

On the other hand, regions such as Somali and Afar may be difficult for health professionals interventions and access due to poor health coverage, limited access to information, and limited health care access (49). In addition to the fact that most of them in these regions are uneducated, they do not know much about contraception. People also might believe that having many children is considered a source of wealth and a symbol of blessing by God. Furthermore, since these people live in rural and desert areas and nomadic lives, it is difficult to intervene and educate them (60).

The strength of this study includes the use of a large sample size. As the sample size increases, the sample gets closer to the actual population, which decreases the potential for deviations from the actual population, and it could be reliable to use by other upcoming researchers. In addition to this, an advanced statistical model that can take the nature of the data into account was employed. However, the present study should be interpreted with several limitations; because of the cross-sectional nature of the study, making causal inferences about the observed associations might not be possible. Moreover, social desirability bias might be introduced as the data were entirely based on self-reports.

## Conclusion

This study revealed that contraceptive usage among married women is comparatively low, with wide regional variation. Women's age, educational status, wealth status, number of children born, antenatal care follow-up, regions, age at first birth, and women from high-level communities were statistically significant variables linked to contraceptive use. The government and other vital actors of maternal and child health stakeholders can facilitate activities, increase knowledge and change attitudes about contraception by supporting and guiding health providers, community leaders, health extension workers in Afar and Somali regions. On the other hand, region-based intervention, mentoring, and funding support may have a favorable influence on contraception coverage in the mentioned regions, including those identified in the spatial study. It is preferable to raise awareness among mothers about ANC, and assist those who are financially disadvantaged or do not have access to health facilities. As a result, public health interventions, particularly those that can improve contraceptive information dissemination and community-level contraceptive utilization rates, are urgently needed at the national level to address potential barriers.

## Data Availability

The original contributions presented in the study are included in the article/Supplementary Material, further inquiries can be directed to the corresponding author.
